# Effect of light sources with and without UVA on selected behavior and health indicators in commercial broiler breeder flocks

**DOI:** 10.1016/j.psj.2023.102927

**Published:** 2023-07-08

**Authors:** G. Vasdal, K.E. Kittelsen, F. Tahamtani

**Affiliations:** Norwegian Meat and Poultry Research Centre, 0515 Oslo, Norway

**Keywords:** comfort behavior, exploration, locomotion, resting

## Abstract

As new light sources are being developed for poultry houses, systematic investigations on how these influence behavior and health in commercial broiler breeders are needed. Therefore, the aim of this study was to investigate the effects of 2 light sources (Evolys with UVA (LED) and Biolux 965 (CFL)) on the behavior and health of 2 broiler breeder hybrids during the production period. Eight commercial breeder flocks (Ross 308 *n* = 4, Hubbard JA757 *n* = 4) with Evolys (Ross *n* = 2, Hubbard *n* = 2) or Biolux (Ross *n* = 2, Hubbard *n* = 2) were visited at 25 and 50 wk of age to record behavior and health. Behaviors included resting, locomotion, exploration, comfort, feather pecking, aggression, and mating, while health was recorded by a transect walk, scoring the number of birds observed with: feather loss (**FL**) on head, back/wings, breast, and tail, wounds on head, back/wings, and tail, dirty plumage, lameness, sickness, and dead birds. The most common behaviors were resting, locomotion, comfort, and exploration, and these were influenced by a 3-way interaction between light source, hybrid, and age. Light source did not affect behavior in Hubbard birds at any age. In contrast, Ross birds housed in Evolys rested less at 50 wk compared to Biolux (*P* = 0.04) and showed more locomotion at 25 wk in Biolux compared to Evolys (*P* < 0.0001). Ross birds at 25 wk explored more in Biolux compared to Evolys (*P* = 0.0007). More comfort behavior was performed in Evolys in 25-wk-old Ross (*P* = 0.002), but not at 50 wk. These inconsistencies might be due to low sample size, which is a limitation in the study. The most common health indicators were FL on back/wings (mean 3.9%), wounds on back/wings (mean 0.22%), and FL head (mean 0.18%), with no effect of light source, hybrid, or age on FL back/wings, breast, or tail, but with increased FL on the head with increased age (*P* = 0.0008). In conclusion, the behavior of Ross birds seemed to be affected by light source, while the Hubbard birds were not. Light source had minor effects on the selected health indicators in the 2 hybrids.

## INTRODUCTION

Light is one of the most important physical factors in commercial poultry production, and the effect of light on poultry behavior and production has been the focus of scientific studies for almost 100 yr (Penquite and Thompson, 1933). Despite decades of studies, there are still important knowledge gaps regarding how light affects behavior and health, especially in breeding poultry (Oso et al., 2022). This may partly be because bird vision differs from human vision, making inferred understanding harder. For example, birds have a wider visible range than humans, including the UV part of the spectrum (Maier, 1992; Osorio et al., 1999; Osorio and Vorobyev, 2008). Furthermore, light perception occurs at 2 sites in birds; in photoreceptors in the retina of the eye, and in extraretinal photoreceptors in the pineal gland, pituitary, and hypothalamus, which mediate circadian rhythm and sexual maturation (Underwood et al., 2001; Kumar et al., 2004).

In most modern poultry facilities, artificial light is the only light source, and different types of artificial light sources have been used over the last decades. The different qualities of light include light source, photoperiod, intensity, and wavelength (color), and each of these qualities have their own impact on broiler behavior and production (Lewis and Morris, 2000). Common light sources today include compact fluorescent lamps (**CFL**) and light-emitting diodes (**LED**), with the latter often being preferred due to their low energy use, long operating life and availability in different wavelengths (Huth and Archer, 2015). Broilers housed under LED lights are reported to be less fearful and have better feed efficiency compared to broilers housed under CFLs (Huth and Archer, 2015). However, as wavelengths can differ between LED bulbs depending on its spectral components, there are conflicting reports on the impact of LED light on poultry performance (Karakaya et al., 2009; Huth and Archer, 2015). Furthermore, LED lights including UVA and UVB wavelengths have been introduced over the last years, where results point to positive effects on behavior, welfare, and productivity (Rana and Campbell, 2021), including improved bone composition, improved egg production (Wei et al., 2019), and reduced fear in caged hens (Sobotik et al., 2020). Studies have shown that both broilers and laying hens prefer light with UV when given a choice (Kristensen et al., 2007; Liu et al., 2018). Presence of UVA also affects mate choice in broiler breeder hens, where females will spend more time inspecting males and being in close proximity to males lit by UVA light (Jones and Prescott, 2000; Jones et al., 2001), likely due to mate choice cues in the males’ plumage being visible under UVA wavelengths.

Broiler breeder farming is a highly specialized and standardized production, where the primary objective includes high-quality fertile eggs, with a high hatchability rate. The breeder flocks consist of females and males, with the male-female ratio of about 1 male to 12 females, depending on the strain. The breeding flocks are usually provided with a littered area and an elevated area with slats and nest boxes. Under natural condition, male aggression toward females is rare, because males and females have their own social hierarchies, where males display elaborate mating behavior and the females then choose which males to mate with (Wood-Gush, 1958). However, in modern breeder flocks, males rarely display courtship behavior and show relatively high levels of aggression toward females, which can result in forced mating and injuries on the females (Millman et al., 2000). This aggressive behavior also leads to the females spending more time on the elevated slats, while the males tend to stay in the littered area (Leone and Estevez, 2008). Whether presence of UVA in the house affects the level of aggression between males and females has previously not been investigated in commercial broiler breeder flocks.

When the breeder pullets arrive at the production facility at around 18 wk, artificial light is used to control the sexual maturation of the birds (Lewis et al., 2010). Longer photoperiods over 12 h activate the reproductive axis, especially through presence of red wavelengths which penetrates the skull and stimulate the extraretinal photoreceptors (Mobarkey et al., 2010). Red wavelengths have positive effects on egg production in broiler breeders (Mobarkey et al., 2010) and on the reproductive traits of broiler breeder roosters (Bartman et al., 2021). On the other hand, green wavelengths may reduce egg production (Mobarkey et al., 2010), while blue wavelengths generally promote growth in young breeder birds (Yang et al., 2016). However, the wavelengths must be considered together with light intensity, as some studies report that wavelengths alone does not affect production parameters in broilers (Franco et al., 2022). Two common light sources in Norwegian broiler breeder flocks are Biolux 965 CFL and a LED light called Evolys consisting of LED diodes and UVA diodes. The light environment created by these light sources in flocks of broiler breeders with either brown (Hubbard) or white (Ross 308) plumage is described in detail by Vasdal et al. (2022a). The light environment was in general found to be relatively similar, with the main difference being more blue-dominated light in the Biolux and more red-dominated light and presence of UV wavelengths in Evolys houses. Plumage color had minimal effect on the light environment. There have been few investigations into the behavioral differences in the Hubbard and Ross parent stocks, and the main known difference between the 2 is the morphology of the female line, where the Hubbard female is a dwarf hen weighing around 2 kg at 60 wk, while the Ross female weighs about 4 kg at 60 wk. The males in both hybrids are relatively similar with regards to color and growth curves, both weighing around 5 kg at 60 wk. Previous studies have found an effect of UVA presence on laying hen behavior, including more active behaviors such as foraging and locomotion (Wichman et al., 2021). As an increasing number of poultry farmers are investing in light sources with UVA, there is a need to know if the presence of UVA result in differences in behavior, mating, and aggression in commercial breeder flocks, and if these effects vary between different hybrids.

A basis for good animal welfare is good health, with absence of lameness and wounds, good plumage, and overall low mortality, but little is known whether different light sources affect health indicators in broiler breeders. In order to assess important health and welfare parameters in large flocks, Vasdal et al. (2022b) developed a transect sampling method for cage-free laying hens, and this method has been found to be a practical, time efficient, and reliable method for on-farm assessment in large flocks of broilers (BenSassi et al., 2019), turkeys (Marchewka et al., 2015), and ducks (Abdelfattah et al., 2020). The method is based on the transect sampling methodology, where an assessor walks through the house along predetermined paths while counting number of birds observed within each welfare indicator category. The method requires no animal handling, it resembles the daily flock checks conducted by farmers and is therefore easy to apply in large commercial flocks.

The aim of this study was to investigate the effects of 2 commonly used light sources for broiler breeders (LED: Evolys with UVA and CFL: Biolux 965) on selected indicators of behavior and health in 2 broiler breeder hybrids during the production period.

## MATERIALS AND METHODS

### Animals and Housing

The study was conducted between March 2022 and April 2023 on 8 commercial broiler breeder flocks (Ross 308 *n* = 4, Hubbard JA787 *n* = 4) located in the eastern and middle parts of Norway. The studied flocks (1 flock/farm) were selected on basis on the light sources in the breeder house. Participation in the study was optional. Each flock consisted of around 7,500 hens and 550 roosters. None of the birds were beak trimmed and in 2 of the flocks the males were toe clipped (1 Hubbard flock and 1 Ross flock). Hubbard hens typically reach 5% production at 23 wk of age and 1,945 g (Hubbard, 2023), while Ross hens reach 5% production at 25 wk and 2,970 g (Aviagen, 2021). Both hybrids followed the feeding scheme according to the breeder manual, with ad lib feeding from the age they reached 5% production. All flocks were visited twice during the production period, at 25 and 50 wk of age. These ages were selected on the background that both hybrids were expected to have reached 5% production at 25 wk, and to observe them again toward the end of the production period to detect potential changes in behavior due to increased age. The houses were between 14 and 22 m wide, covering a floor area of 1,134 to 1,606 m^2^ (Table 1), and all had concrete floor with wood shavings, automatic feeder and drinker lines, elevated slats and nest boxes, and mechanical ventilation keeping the temperature around 20°C. The flocks were managed according to standard practices with regards to feed, water, and litter (Norwegian Quality Standard; KSL, 2021). The roosters arrived at the farm at 17 wk and the hens at 18 wk. All flocks were depopulated after 60 wk of age.

**Table 1:** Details of light quality and floor area in the 8 broiler breeder houses (mean 25 and 50 wk of age) with either Biolux 965 CFL (compact fluorescent lighting) or Evolys with UVA.

Flock	Light source	Hybrid	Area (m^2^)	Stocking density (birds/m^2^)	No. light sources	Lux	Photoperiod (h)	Kelvin^1^	CRI^2^	Flicker index^3^	Htz^4^
1	Biolux 965	Ross 308	1425	5.6	72 CFL	11.1	13	5262.3	93.8	0.01	100
2	Biolux 965	Ross 308	1440	5.5	70 CFL	7.2	13	5190.1	94.1	0.01	100
3	Biolux 965	Hubbard	1134	6.7	48 CFL	25.3	15	5760.5	93.2	0.01	100
4	Biolux 965	Hubbard	1275	6.3	66 CFL	24.0	15	5953.1	91.5	0.01	100
5	Evolys	Ross 308	1491	5.5	48 LED, 11 UVA	14.1	13	4725.2	84.3	0.05	400
6	Evolys	Ross 308	1606	5.1	48 LED w/ UVA	11.0	13	4251.0	83.0	0.02	400
7	Evolys	Hubbard	1134	6.6	36 LED, 9 UVA	22.2	15	4138.4	86.1	0.03	400
8	Evolys	Hubbard	1230	6.3	42 LED, 6 UVA	30.8	15	4742.8	86.2	0.02	400

1 Color temperature of the light source.

2 Color rendering index, the effect of a light source on the color appearance of objects in comparison with a natural light source.

3 A measure of the quantity of light at high intensity against the quantity of light at low intensity over 1 cycle.

4 Number of oscillations of a light cycle in 1 s.

With regards to the artificial light in the breeder house, 4 of the flocks (Ross *n* = 2, Hubbard *n* = 2) had Evolys light (LED with UVA (Type E21, Evolys, Oslo)) while 4 of the flocks (Ross *n* = 2, Hubbard *n* = 2) had Osram Biolux 965 (Munich, Germany). None of the houses had windows. Further details of the house, photoperiod, and light sources are presented in Table 1.

Because the study involved no experimental manipulations or invasive procedures, it was exempt from approval of animal use by the Norwegian Food Safety Authority (Norwegian Regulations on Use of Animals in Research, 2015).

### Light Quality Recordings

The light quality in each house was recorded using a spectrometer (UPRTEK MK 350S Premium, Elma Instruments, Oslo, Norway) in the same 2 locations in all houses, at animal height; in the middle of the litter area and in the middle of the slatted area. The recordings included light illuminance (lux), color temperature of the light source (**CCT**, expressed in kelvin, K), color rendering index (**CRI**, the effect of a light source on the color appearance of objects in comparison with a natural light source), flicker index (a measure of the quantity of light at high intensity against the quantity of light at low intensity over 1 cycle), and the number of oscillations of a light cycle in 1 s (hertz, htz). Mean values for each quality at wk 25 and 50 were then calculated per house (Table 1). The light intensity varied between houses from 7 lux up to 30 lux, and the 4 Hubbard flocks had the highest light intensities. In general, the Biolux houses had a higher color temperature (i.e., colder white) compared to Evolys. Furthermore, the spectral outcome in houses with Biolux consisted of 2 lower peaks at 450 nm (blue) and 500 nm (blue) and 2 higher peaks at 550 nm (green) and 620 nm (orange). The Evolys produces one sharp peak at 400 nm (violet) and a lower peak at 450 nm (blue). Further details of the light environment produced by these light sources can be found in Vasdal et al. (2022a).

### Behavioral Observations

At 25 and 50 wk of age, the behavior of the birds was scored by a single observer using direct observation in 4 different areas of the house in the morning (2–4 h after light was turned on). The location of each patch was chosen randomly, however making sure to include different areas of the house such as litter area, near walls, center of house, and elevated slats. Each area consisted of an observation patch of approximately 3 m^2^. The width of the patch was defined by the feeder lines while the length was defined by structures in the elevated slats, which were evenly distributed along the length of the house. After entering the broiler house, the observer walked slowly through the flock for about 10 min to allow the animals to get used to the observer's presence and resume ongoing activities. After reaching a new observation patch, the observer stood still for 10 min before observations started. The observer was standing several meters away from the observation patch, in order to reduce the risk of disturbing the birds. The number of birds performing each of the behaviors in Table 2 was scored every 2 min over a 20-min period per patch, for a total of 40 registrations/flock/age. Birds performing other behavior such as feeding and drinking were not included.

**Table 2:** Ethogram of the 12 behaviors scored in each flock.

Each second minute	Number of birds observed performing the behavior
Resting	Sitting, lying
Locomotion	Walking, running, jumping,
Exploration	Ground pecking, scratching
Perching	Sitting on a structure, keel in contact with structure
Dustbathing	While lying with fluffed feathers, bird simultaneously and rapidly lifts the wings up and down multiple times; scooping loose substrate material up into the feathers
Comfort behavior	Body shake, wing flapping, stretching wings or legs, preening itself
Feather pecking	Pecking the feather of other birds, with or without feathers pulled out
Aggression male–male	Male forcefully pecking another male/hopping toward/threatening
Aggression male–female	Male forcefully pecking a female/hopping toward/threatening
Aggression female–male	Female forcefully pecking a male/hopping toward/threatening
Aggression female–female	Female forcefully pecking another female/hopping toward/threatening
Mating behavior	Male mates with a female

### Health Assessment

Following the method of Vasdal et al. (2022b), standardized transect walks were made along the full length of the house to record the number of birds observed per transect that were showing each of 12 predefined welfare indicators (Table 3). The transect was done after the behavior observations were finished. All indicators are scored on a binary scale, focusing on the presence or absence of relatively severe rather than mild cases, which minimizes the risk of omitting birds. The observations always started with the left wall transect. When reaching the other end of the house, the observer returned collecting data in the next transect. This process was repeated in houses with more than 2 transects. While walking along each transect, stops were made as needed to allow assessment of birds on the litter area as well as on the elevated slatted area. Birds in nest boxes were observed by opening the curtains on approx. a third of them.

**Table 3:** Description of 12 welfare indicator categories assessed by the Aviary Transect method (as in Vasdal et al., 2022b).

Transect	Description
FL head	Missing feathers on head and neck ≥5 cm in diameter (equals “c” score in WQ)
FL back/wing	Missing feathers on back and/or wings ≥50% of the back
FL breast	Missing feathers on breast ≥50% of breast
FL tail	Missing or clearly damaged feathers on the tail, including broken or torn feathers
Dirty	Very clear and dark staining of the back, wing, and/or tail feathers of the bird, covering at least 30% of the body area
Wounds head	Bird has visible marks on the head, beak, or neck related to fresh or older wounds. Peck injuries comb
Wounds back/wing	Bird has visible marks on the back or wings related to fresh or older wounds
Wounds tail	Bird has visible marks on the tail related to fresh or older wounds
Lameness	Bird has clearly difficulty walking, use of wing for support when walking
Sick	Bird showing clear signs of impaired health
Dead	Dead birds found during the transect

### Statistical Analysis

Statistical analyses were performed using the software SAS 9.4 (SAS Institute Inc., Cary, NC; SAS Institute Inc., 2016). The number of birds per scan performing each behavior in the ethogram were summed and averaged across light sources, hybrids, and ages. With the data collected during the health transect, the frequency of birds with each welfare indicator was calculated as a proportion of the total estimated number of birds in the flock. The data from the direct behavioral observations and health transect were analyzed using the mixed procedure, with the fixed factors light source, hybrid, and age as well as their interactions. When appropriate, insignificant interactions were removed from the model in a backward inclusion method until the final model contained only the individual fixed factors. For the behavior variables, observation patch nested in flock was included in the model as a random effect. For the health variables, only flock was included as a random effect. When necessary, post hoc analyses were performed with the Tukey test (Tukey's HSD test). The following behaviors had very low occurrence and so could not be analyzed: dust bathing, feather pecking, mating, perching, and all forms of aggression. Descriptive statistics are presented for these behaviors instead. Likewise, the prevalence of sick and dirty birds was too low to be analyzed.

## RESULTS

### Behavioral Observations

There was too low occurrence of aggression, dust bathing, feather pecking, mating, and perching behavior to include in the analyses, thus only descriptive statistics are presented (Table 4). The average number of birds per scan per observation patch was 22.22 (range: 7–58).

**Table 4:** Descriptive statistics (mean and std. dev.) of the number of birds engaged in the behaviors not included in the analyses across light sources, hybrids, and ages.

		Biolux				Evolys			
		Hubbard		Ross		Hubbard		Ross	
Behavior	Age (wk)	25	50	25	50	25	50	25	50
Aggression FF^1^	Mean	0.03	0.20	0.08	0.11	0.01	0.03	0.31	0.16
	Std. dev.	0.16	0.40	0.27	0.32	0.11	0.16	0.54	0.51
Aggression FM^1^	Mean	0.01	0.55	0.00	0.05	0.00	0.18	0.05	0.05
	Std. dev.	0.11	0.88	0.00	0.22	0.00	0.38	0.22	0.27
Aggression MF^1^	Mean	0.16	0.08	0.08	0.03	0.05	0.01	0.04	0.05
	Std. dev.	0.37	0.27	0.27	0.16	0.22	0.11	0.19	0.22
Aggression MM^1^	Mean	0.28	0.01	0.31	0.01	0.04	0.00	0.03	0.00
	Std. dev.	0.45	0.11	0.52	0.11	0.19	0.00	0.16	0.00
Dust bathing	Mean	0.68	0.00	0.58	0.94	0.85	0.00	3.91	0.16
	Std. dev.	2.06	0.00	1.65	1.16	2.24	0.00	2.05	0.60
Feather pecking	Mean	0.26	0.23	0.05	0.03	0.00	0.10	0.50	0.09
	Std. dev.	0.52	0.45	0.22	0.16	0.00	0.30	0.66	0.33
Mating	Mean	0.38	0.04	0.79	0.10	0.09	0.01	0.54	0.11
	Std. dev.	0.49	0.19	0.69	0.30	0.28	0.11	0.71	0.39
Perching	Mean	3.88	0.69	0.00	0.00	0.78	0.00	0.24	0.00
	Std. dev.	2.86	1.06	0.00	0.00	2.21	0.00	0.51	0.00

1 FF = aggression between females. MM = aggression between males. FM = females being aggressive toward males. MF = males being aggressive toward females. *N* = 80.

### Resting

**Figure 1:**
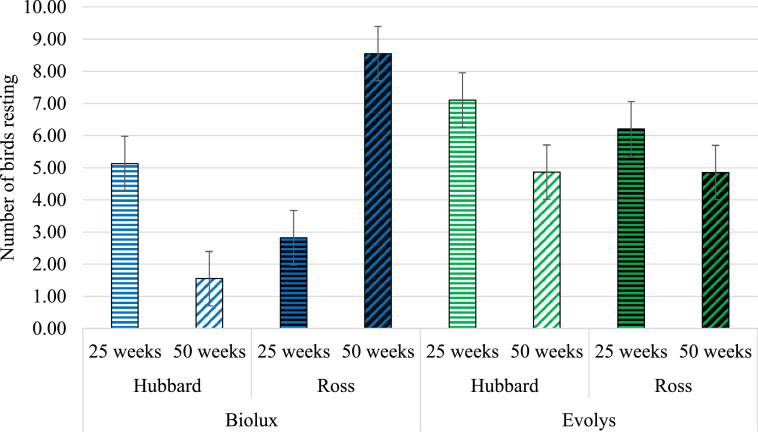
Number of resting birds (LS means ± SE) in the hybrids and ages in Biolux or Evolys.

There was a 3-way interaction of the effects of hybrid, light source, and age on resting behavior (*F*_1,603_ = 90.82; *P* < 0.0001). Light source did not affect the resting behavior of Hubbard birds at 25 (*P* = 0.71) or 50 wk of age (*P* = 0.11). In contrast, 50-wk-old Ross birds rested more under Biolux compared to Evolys lighting (*P* = 0.04), while at 25 wk of age, Ross birds tended to rest more under Evolys (*P* = 0.091) (Figure 1).

Age affected resting behavior in Hubbard birds, as they rested more at 25 wk compared to 50 wk, both when exposed to Biolux (*P* < 0.0001) and Evolys (*P* < 0.0001). Ross birds exposed to Evolys rested more at 25 wk compared to 50 wk (*P* = 0.047), while Ross birds exposed to Biolux rested more at 50 wk of age compared to 25 wk (*P* < 0.0001) (Figure 1). At 25 wk, there was no difference in resting behavior between the hybrids whether they were exposed to Biolux (*P* = 0.53) or Evolys (*P* = 0.99). No difference was found between the hybrids at 50 wk of age when exposed to Evolys (*P* = 1.00). However, when exposed to Biolux, 50-wk-old Hubbard birds rested significantly less than 50-wk-old Ross birds (Figure 1).

### Locomotion

**Figure 2:**
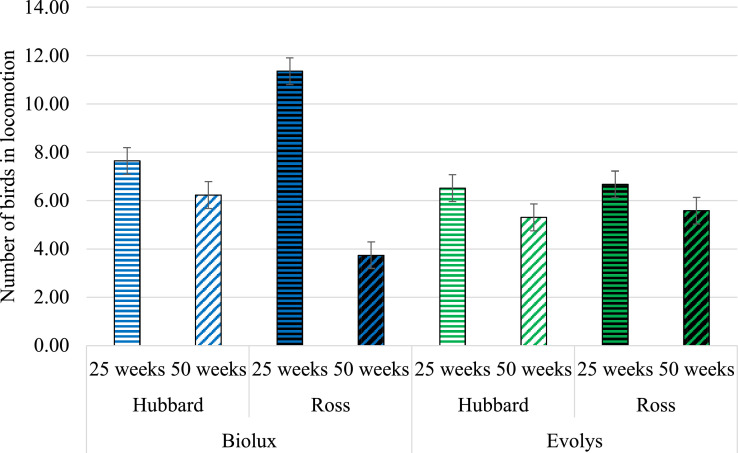
Number of birds showing locomotion (LS means ± SE) in the 2 hybrids and ages in Biolux or Evolys, respectively.

There was a 3-way interaction of the effects of hybrid, light source, and age on locomotion (*F*_1,604_ = 72.76; *P* < 0.0001). The type of light source did not affect locomotion of Hubbard birds at 25 (*P* = 0.84) or at 50 wk of age (*P* = 0.94). Neither did it affect Ross birds at 50 wk (*P* = 0.27). However, at 25 wk, Ross birds showed more locomotion in Biolux compared to Evolys light (*P* < 0.0001) (Figure 2).

Under Biolux exposure at 25 wk of age, locomotion was higher in the Ross birds compared to Hubbard birds (*P* < 0.0001). However, at 50 wk, locomotion was higher in the Hubbard birds compared to Ross birds when exposed to Biolux (*P* = 0.03) (Figure 2). When exposed to Evolys, however, no differences between the hybrids were found at 25 wk (*P* = 1.00) or at 50 wk of age (*P* = 1.00). In general, more locomotion was observed when the birds were 25 wk of age compared to 50 wk, for both hybrids and under exposure to either light source (Hubbard under Biolux: *P* = 0.004; Ross under Biolux: *P* < 0.0001; Hubbard under Evolys *P* = 0.025; Ross under Evolys: *P* = 0.07) (Figure 2).

### Comfort Behavior

**Figure 3:**
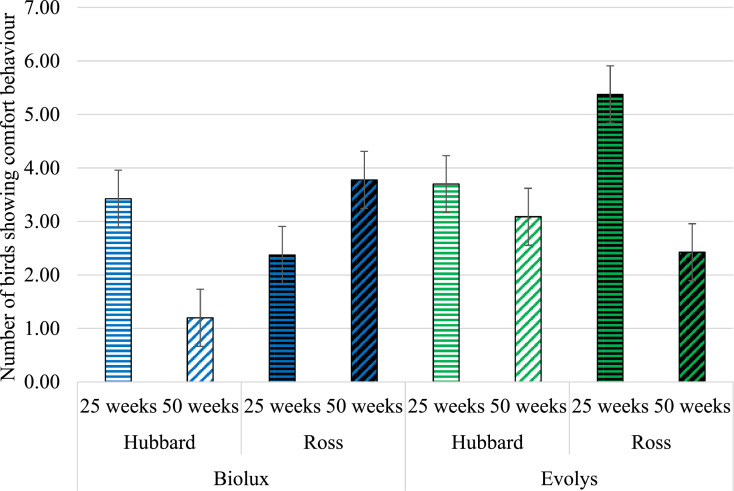
Number of birds showing comfort behavior (LS means ± SE) in the 2 hybrids and ages in Biolux or Evolys, respectively.

There was a 3-way interaction of the effects of hybrid, light source, and age on the performance of comfort behavior (*F*_1,604_ = 130.10; *P* < 0.0001). Within age, light source only affected comfort behavior in Ross birds at 25 wk of age, with more comfort behavior being performed under Evolys compared to Biolux (*P* = 0.002). This effect of light was not seen for Ross birds at 50 wk (*P* = 0.63), or for Hubbard birds at 25 or 50 wk (*P* = 1.00 and *P* = 0.20, respectively) (Figure 3).

Ross birds performed more comfort behavior compared to Hubbard birds at 50 wk of age under Biolux lighting (*P* = 0.016) but not at 25 wk (*P* = 0.86). There was no hybrid difference under Evolys lighting when the birds were 25 or 50 wk (*P* = 0.34 and *P* = 0.99, respectively). Under Biolux exposure, Hubbard birds performed more comfort behavior at 25 compared to 50 wk (*P* < 0.0001). In contrast, comfort behavior was more prevalent in Ross birds at 50 wk compared to 25 when exposed to Biolux light (*P* < 0.0001). Under Evolys lighting, Ross birds performed more comfort behavior at 25 compared to 50 wk (*P* < 0.0001), while no differences across age were observed for the Hubbard birds (*P* = 0.27) (Figure 3).

### Exploratory Behavior

**Figure 4:**
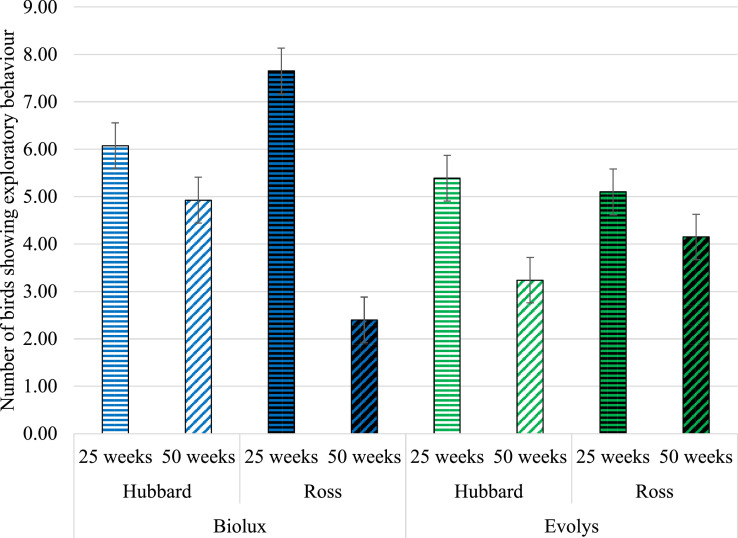
Number of birds showing exploratory behavior (LS means ± SE) in the 2 hybrids and ages in Biolux or Evolys, respectively.

There was a 3-way interaction of the effects of hybrid, light source, and age on the performance of exploratory behavior (*F*_1,604_ = 78.31; *P* < 0.0001). Ross birds at 25 wk explored more under Biolux compared to Evolys lighting (*P* = 0.0007) but did not differ at 50 wk (*P* = 0.17). Hubbard birds, in contrast, did not differ in exploratory behavior between Biolux and Evolys lighting at 25 (*P* = 0.84) or 50 wk (*P* = 0.21) (Figure 4).

Within age, Ross birds in Biolux explored more at 25 wk (*P* = 0.02) and less at 50 wk (*P* = 0.005) compared to Hubbard birds. Under Evolys lighting, however, the 2 hybrids did not differ from each other at 25 or at 50 wk (*P* = 0.99 and *P* = 0.88, respectively). Under both types of light sources, exploration went down from 25 to 50 wk for both hybrids (*P* < 0.03).

### Health Indicators

The descriptive statistics for the selected health indicators are presented in Table 5, and the most common health indicators were feather loss (**FL**) on the back/wings (mean 3.9%), wounds on back/wings (mean 0.22%), and FL head (mean 0.18%). There was no effect of hybrid (*F*_1,12_ = 1.10; *P* = 0.31), age (*F*_1,12_ = 1.19; *P* = 0.29), or light source (*F*_1,12_ = 1.10; *P* = 0.31) on FL on the back/wings. There was no effect of hybrid or light source on FL head (*F*_1,5_ = 0.47; *P* = 0.52 and *F*_1,5_ = 0.22; *P* = 0.66), but FL head increased with age (25 wk LS means ± SE: 0.003 ± 0.01%; 50 wk LS means ± SE: 0.06 ± 0.01%; *F*_1,7_ = 13.60; *P* = 0.008). Furthermore, FL breast was not affected by light source (*F*_1,5_ = 0.69; *P* = 0.44), hybrid (*F*_1,5_ = 0.69; *P* = 0.44), or age (*F*_1,7_ = 1.78; *P* = 0.22). Finally, FL tail was also not affected by hybrid (*F*_1,5_ = 0.14; *P* = 0.72), light source (*F*_1,5_ = 0.43; *P* = 0.54), or age (*F*_1,7_ = 4.82; *P* = 0.06).

**Table 5:** Flock prevalence descriptive statistics (mean, std. dev., min, max) for the assessed health parameters in the 8 broiler breeder flocks.

Health parameter	Mean	Std. dev.	Min	Max
Flock prevalence (%)				
Dead	0.00	0.01	0.00	0.04
Dirty	0.00	0.00	0.00	0.00
FL^1^ back/wings	3.90	14.46	0.00	58.07
FL^1^ head	0.18	0.60	0.00	2.43
FL^1^ breast	0.08	0.24	0.00	0.97
FL^1^ tail	0.17	0.30	0.00	1.04
Lameness	0.02	0.02	0.00	0.07
Sick	0.01	0.01	0.00	0.04
Wounds back/wings	0.22	0.28	0.00	0.98
Wounds head	0.04	0.05	0.00	0.17
Wounds tail	0.03	0.06	0.00	0.21

1 FL = feather loss. *N* = 16.

The prevalence of lameness was affected by the interaction between light source and age (*F*_1,6_ = 14.99; *P* = 0.008). At 25 wk, more birds housed in Biolux light were visibly lame (LS means ± SE: 0.05 ± 0.009%) compared to those housed in Evolys light (LS means ± SE: 0.006 ± 0.009%; *P* = 0.03). In addition, the prevalence of lameness was higher at 25 wk compared to at 50 wk for the flocks exposed to Biolux light (LS means ± SE: 0.016 ± 0.009%; *P* = 0.02) whereas no difference between ages was detected for the flocks exposed to Evolys light (Evolys 50 wk LS means ± SE: 0.017 ± 0.009; *P* = 0.64). No other significant pairwise comparisons were found. Furthermore, there was no overall effect of hybrid on the prevalence of lameness (*F*_1,5_ = 0.25; *P* = 0.63).

There was an effect of the interaction between hybrid and age on the prevalence of wounds on the back and wings (*F*_1,6_ = 15.80; *P* = 0.007). Hubbard birds at 50 wk had a higher prevalence of these wounds (LS means ± SE: 0.60 ± 0.09%) compared to younger Hubbard birds (LS means ± SE: 0.02 ± 0.09%; *P* = 0.01) and Ross birds of the same age (LS means ± SE: 0.09 ± 0.09%; *P* = 0.03) and tended to have a higher prevalence compared to 25-wk-old Ross birds (LS means ± SE: 0.17 ± 0.09%; *P* = 0.06). As for head wounds, there was no effect of light source (*F*_1,5_ = 0.28; *P* = 0.62), hybrid (*F*_1,5_ = 0.67; *P* = 0.45), or age (*F*_1,7_ = 0.8; *P* = 0.40). Likewise, there was no effect of any of the fixed factors on the prevalence of wounds on the tail (light source: *F*_1,12_ = 1.55; *P* = 0.23; hybrid: *F*_1,12_ = 2.77; *P* = 0.12; age: *F*_1,12_ = 3.33; *P* = 0.09). Finally, there was no effect of light source (*F*_1,12_ = 1.52; *P* = 0.24), hybrid (*F*_1,12_ = 0.33; *P* = 0.57), or age (*F*_1,12_ = 1.72; *P* = 0.21) on the prevalence of dead birds observed during the health transect.

## DISCUSSION

The aim of this study was to investigate the effects of 2 commonly used light sources for broiler breeders (LED: Evolys with UVA and CFL: Biolux 965) on the behavior and health in 2 broiler breeder hybrids during the production period. Only 2 flocks per light source and hybrid were included in the study, which is a limitation of the study and thus the results must be interpreted with caution. The small sample size could also be the cause of some of the inconsistent results. Nevertheless, the results indicate that the behavior of Hubbard birds is less affected by light source compared to Ross birds.

Based on previous studies on aggression in broiler breeder males (e.g., Millman et al., 2000), we expected to observe aggression between the birds, and that presence of UVA could reduce aggression, especially between males and females due to reduced stress levels (Rana and Campbell, 2021) and mate choice cues being visible under UVA wavelengths (Jones and Prescott, 2000; Jones et al., 2001). However, observations of aggression between the birds were rare at both 25 and 50 wk of age, regardless of hybrid and light source. The same was true for dust bathing, perching, feather pecking, and mating, which could not be included in the analyses due to very low occurrence across the flocks. The low occurrence of these behaviors could partly be due to time of observations. Peak dust bathing is reported to be around 6 h after lights-on (Vestergaard, 1982), while the flocks in this study was observed 2 to 4 h after lights-on. Perching, on the other hand, is reported in broiler breeders to be low during the light period (Brandes et al., 2020) in both Ross and Hubbard breeders (Vasdal et al., 2022c). Increased mating frequency is observed in the evening compared to the morning in broiler breeders (e.g., Bilcik and Estevez, 2005), which may help explain the low mating frequency observed in the present study. A study in 8 commercial breeder flocks observed mating activity and aggression during the last hours before lights-out and found higher prevalence of both behaviors than in the present study (de Jong et al., 2009). Thus, observations of mating and aggression should include observations during the last hours before light-out.

The most frequently observed behaviors across the flocks were resting, locomotion, comfort, and exploration, and these were influenced by a 3-way interaction between light source, hybrid, and age. The prevalence of these behaviors in Hubbard flocks was not affected by light source at any age. Light source affected resting behavior in Ross birds, which rested more at 25 wk when housed in Evolys and more at 50 wk when housed in Biolux. There are no published systematic investigations of the behavioral differences in the Hubbard and Ross broiler breeders, but as the Hubbard hens reach 5% production at 23 wk and 1,945 g, while Ross hens reach 5% production at 25 wk and 2,970 wk, live weight, age of maturity, and onset of lay could be the reason behind some of the present results. Lighter hybrids are generally considered more active, both in laying hens (Kozak et al., 2016) and broiler breeders (Gebhardt-Henrich et al., 2018), and we expected to observe more resting in the heavier Ross birds compared to Hubbard birds. This effect could be further enhanced due to the higher light intensities in the 4 Hubbard houses, as light intensity is known to stimulate activity in poultry (e.g., Lewis and Morris, 2000). However, when housed in Biolux, Hubbard birds rested more than Ross at 25 wk, while Ross rested more at 50 wk. Furthermore, we expected the birds to rest more with age, but Hubbard birds rested more at 25 wk than at 50 wk, regardless of light source. The Ross birds showed an inconsistent trend, with more resting behavior at 50 wk in Biolux, but not in Evolys. The reasons behind these results are unclear, and few studies have described the behavior patterns or time budgets of commercial broiler breeders during the production period. However, studies focusing on perching behavior report a reduction in mobility and perching with increased age in Ross birds (Mens and van Emous, 2022), possibly due to increased body weight and reduced mobility, which could have led to increased resting with age in some of the Ross flocks, and not in the lighter Hubbard flocks.

Based on previous studies in laying hens, we could expect a higher activity level when UVA was present (Wichman et al., 2021). Contrary to this, locomotory behavior, such as walking, running, and jumping, was not affected by light source in the Hubbard flocks at any age, while the Ross flocks showed more locomotion in Biolux at 25 wk compared to Evolys, and with no differences between the light sources at 50 wk. The effect of UVA on behavior depends on the intensity, and a study by Rana et al. (2021) found more foraging, preening, and ground pecking in the lower intensities of UVA/B. Thus, the level of UVA needed to affect behavior in broiler breeders must be further studied, as the effects likely also vary between hybrids. We expected more locomotion in the lighter Hubbard, but across the 4 Biolux flocks, Ross flocks showed more locomotion at 25 wk, while Hubbard flocks showed more locomotion at 50 wk. There were no differences in locomotion between hybrids across the Evolys flocks. In general, more locomotion was observed at 25 wk compared to 50 wk, which is in accordance with studies in laying hens that found a reduction in activity with increasing age (Kozak et al., 2016).

Comfort behaviors such as preening, body shake, and wing flapping are considered positive behavior indicators (e.g., Vas et al., 2023), and an increased prevalence of these behaviors could indicate reduced levels of stress and fearfulness in the flock. As with resting and locomotion, the prevalence of these behaviors was not affected by light source across the Hubbard flocks. Light source affected the Ross flocks, with more comfort behavior observed at 25 wk in Evolys, and more at 50 wk in Biolux. The lack of consistency in the Ross flocks is interesting, as we could expect more preening in Evolys with UVA present compared to Biolux at both ages. These results further emphasize the need for more studies on the effects of UVA on behavior in different breeder hybrids. There were no differences in amount of comfort behaviors between the hybrids in Evolys, but across the Biolux flocks, Hubbard birds showed more comfort behavior at 25 wk, while Ross birds showed more comfort behavior at 50 wk. The Hubbard birds showed a consistent level of comfort behavior across ages, while Ross birds showed reduced amount of comfort behavior with increasing age, which potentially could be caused by their higher live weight and reduced mobility.

Exploratory behavior, including ground pecking and scratching, is also considered a positive welfare indicator (Vas et al., 2023). Light source did not affect the prevalence of exploration in the Hubbard flocks at any age. Ross birds explored more at 25 wk in Biolux, but there were no differences between light sources at 50 wk, which is contrary to previous studies in laying hens (e.g., Rana et al., 2021). There were no differences between the hybrids in Evolys, but across the Biolux flocks, Ross birds explored more at 25 wk and less at 50 wk compared to Hubbard birds. With age, the level of exploration was reduced in both hybrids, which is consistent with the findings for locomotory behavior and in accordance with studies in laying hens that reports a reduction in activity with increasing age (Kozak et al., 2016).

The selected health indicators were assessed using a health transect, which has previously been validated for broilers (BenSassi et al., 2019), turkeys (Marchewka et al., 2015), ducks (Abdelfattah et al., 2020), and cage-free laying hens (Vasdal et al., 2022b). The method requires no animal handling, takes approx. 20 min depending on the flock size, and is easy to apply in large commercial flocks. FL on different parts of the body was the most common welfare indicator observed across flocks and ages. There were no effect of light source, hybrid, or age on FL back/wings, FL breast, or FL tail. However, we found increased levels of FL head with increased age. An intact plumage is important for bird comfort, and to protect the females from scratches from the males during mating (de Jong and Guemene, 2011). Across flocks, 3.9% of the birds were scored with FL on the backs, and some studies point to this being caused by mating activity (de Jong and Guemene, 2011). However, a study by Moyle et al. (2010) indicates that FL on the back of the hens is not a good indicator for mating activity. The roosters were toe clipped in only 2 of the 8 flocks in the current study, and the flock with the highest prevalence of wounds on the back and wings (mean 0.98) had toe clipped roosters. Toe clipping is carried out in most countries to prevent feather and skin damage on the hens during mating, but the current results suggest that wounds on the females are still an issue even when roosters are toe clipped. We do not know if the observed wounds on the back and wings were due to males, but as 0.22% of the birds were observed with these wounds, this is a welfare concern. Wounds on the head and tail were less common, with 0.04 and 0.03% of the birds scored with these wounds. Light source did not influence the prevalence of wounds observed, and wounds were in general not affected by hybrid or age. However, Hubbard birds had increasingly more wounds on the back and wings with age, and more wounds than Ross birds at same age. The greater sexual dimorphism in Hubbard compared to Ross could be expected to reduce the amount of wounds, as some studies report that in these lines, the males tend to stand on the ground during mating, rather than on the hen (Gebhardt-Henrich et al., 2018). The causes behind these wounds must be studies further in order to find preventive measures, as the farmers report this to be one of the most pressing issues, both with regards to animal welfare, economy, and public perception.

The prevalence of lameness was overall low across flocks, with 0.02% of the birds scored as lame, and with no differences between hybrids. At 25 wk, more birds housed in Biolux light were visibly lame compared to those housed in Evolys light, while no difference between ages was detected for the Evolys flocks. However, it is difficult to explain why light source would affect lameness without knowing the causation behind the observed lameness. Previous studies have found positive effects of UVB on leg health in broilers (Rana and Campbell, 2021), but to our knowledge, presence of UVA does not. The observed lameness is likely caused by other factors than the light source. Possible explanations for the observed lameness are infectious diseases, like femoral head necrosis, osteomyelitis or arthritis, or other leg pathologies like tendon ruptures.

In conclusion, several of the selected behaviors and health indicators were influenced by a 3-way interaction between light source, hybrid, and age, and with only 2 flocks per light source and hybrid included, which is a limitation in the study and the results must therefore be interpreted with caution. Generally, behavior in Ross birds was affected by light source, while behavior in the Hubbard birds was not. The lighter Hubbard birds rested more with age, while Ross birds rested more with age only in when housed in Biolux. Both hybrids showed reduced locomotion and exploration with age, with Hubbard birds showing more locomotion at 50 wk when housed in Biolux compared to Evolys. Hubbard birds showed a consistent level of comfort behavior across ages, while comfort behavior was reduced with age in the Ross birds. FL and wounds were the most common welfare issue, and there were no observed effects of light source, hybrid or age on these indicators, except increased levels of FL on the head with increased age. The present results suggest that both light sources proved acceptable light qualities for the 2 broiler breeder hybrids. In order to obtain more secure results, a larger sample size and observations at different times during the light period must be included in future studies.
